# Efficient second-harmonic generation of quasi-bound states in the continuum in lithium niobate thin film enhanced by Bloch surface waves

**DOI:** 10.1515/nanoph-2023-0886

**Published:** 2024-03-13

**Authors:** Yun Lin, Yong Ye, Ziliang Fang, Bingyu Chen, Haoran Zhang, Tiefeng Yang, Yuming Wei, Yunxia Jin, Fanyu Kong, Gangding Peng, Hongchao Cao, Heyuan Guan, Huihui Lu

**Affiliations:** Guangdong Provincial Key Laboratory of Optical Fiber Sensing and Communications, Jinan University, Guangzhou 510632, China; Laboratory of Information Optics and Opto-Electronic Technology, Shanghai Institute of Optics and Fine Mechanics, Academia Sinica, Shanghai 201800, China; School of Electrical Engineering and Telecommunications, University of New South Wales, Sydney 2052, NSW, Australia

**Keywords:** Bloch surface wave (BSW), lithium niobate (LiNbO_3_ or LN), bound states in the continuum (BIC), second-harmonic generation (SHG), conversion efficiency (CE)

## Abstract

Nonlinear optics has generated a wide range of applications in the fields of optical communications, biomedicine, and materials science, with nonlinear conversion efficiency serving as a vital metric for its progress. However, the weak nonlinear response of materials, high optical loss, and inhomogeneous distribution of the light field hamper the improvement of the conversion efficiency. We present a composite grating waveguide structure integrated into a Bragg reflector platform. This design achieves high Q in the spectral range by exploiting the unique properties exhibited by the bound states in the Bloch surface wave-enhanced continuum, and efficient second-harmonic generation by close-field amplification with the optical field tightly localized in a nonlinear material. By manipulating the symmetry of the grating, a precise tune over the near field within a designated wavelength range can be achieved. Specifically, we select a photonic crystal configuration supporting surface waves, employing TE polarization conditions and an asymmetry factor of −0.1 between the composite gratings. This configuration resonates at a fundamental wavelength of 783.5 nm, exhibiting an impressive *Q*-factor of 10^6^. Notably, at an incident light intensity of 1.33 GW/cm^2^, we achieve a normalized electric field strength of up to 940 at the fundamental frequency and a second-harmonic conversion efficiency of up to 6 × 10^−3^, significantly amplifying the second-harmonic response. The proposed configuration in this investigation has the potential to be integrated into the field of nonlinear optics for sensing frequency conversion applications.

## 1 Introduction

Nonlinear optics [1–4] has witnessed remarkable progress in recent years across diverse domains, including biomedicine [5], harmonic generation [6], [7], and optical switches [8]. Among the intriguing phenomena in this field is second-harmonic generation (SHG), which unveils the intricate nonlinear optical characteristics exhibited by matter and holds profound significance for its deeper comprehension and exploration. SHG, a frequency-doubling effect, arises from the interaction between incident light and a nonlinear material, resulting in the conversion of two photons with frequency *ω* into a single photon with frequency 2*ω* [9]. This nonlinear phenomenon stems from the interplay between the electrons in the nonlinear material and the optical field. SHG finds extensive utility in optical communications [10], [11], microscope imaging [12], and optical frequency combs [13], [14]. The primary objective of SHG investigations was initially centered on enhancing the response through an augmentation of the waveguide’s coherence length. LiNbO_3_, a crystalline substance renowned for its nonlinear optical characteristics [15], has emerged as a prominent player in the realm of periodically polarized lithium niobate (PPLN) waveguide [16] advancements. Nonetheless, the susceptibility of PPLNs to optical impairment under the influence of intense laser inputs remains a pressing concern. Moreover, these structures typically possess dimensions on the millimeter scale [17], [18], posing significant integration challenges on chip platforms.

In recent years, the investigation into micron and nanostructures has emerged as a thriving domain of scientific inquiry, propelled by the substantial merits offered by micron and nanotechnology, including exceptional precision [19], exemplary performance [20], and scalability [21]. The fact that micron and nanostructures are more suitable for chip integration than millimeter-scale structures has made the field even more compelling. The phenomenon of second-order nonlinear polarization reveals a positive correlation between the strength of the second-order nonlinear signal and the square of the electric field strength at the fundamental frequency [22]: $P^{\left(2\right)}\propto \varepsilon_{0}\chi^{\left(2\right)}\cdot E^{2}$ . Building upon this theoretical foundation, researchers have made notable strides in the fabrication of nanostructures, harnessing the surface plasmon resonance effect [23], the nanoantenna effect [24], or bound states in the continuum [25] to amplify localized field strengths and thereby enhance conversion efficiency. However, the potential of surface plasmon resonance is inhibited by the limitations imposed by metallic materials [26], plagued by ohmic losses and rapid electromagnetic field decay. Similarly, the nanoantenna effect is susceptible to size and directional factors [27], and the coupling process itself is intricate. In stark contrast, dielectric-based BICs have garnered considerable attention owing to their inherent all-dielectric structure, which effectively mitigates losses and endows them with remarkable attributes such as ultra-narrow resonance bandwidth [28], high *Q*-factor [29], and tunability [30]. BICs can be classified into two distinct operating modes: symmetric protected and symmetric unprotected [31], [32]. However, it is imperative to note that BICs exist solely under ideal conditions and are practically imperceptible. To surmount this limitation, researchers have endeavored to generate quasi-BICs by perturbing the structure’s symmetry or manipulating the excitation field [33]–[35]. The extraordinary nonradiative properties intrinsic to BICs have been harnessed across diverse structural frameworks [33], [36]–[38], yielding promising outcomes.

In the selection of nonlinear materials, researchers have focused on the remarkable attributes of LN, including a high nonlinear coefficient (*d*_33_ = 33 pm/V) [39], a wide optical transparency window (350 nm–5 µm) [40], and excellent optical stability. Currently, extensive research has been conducted in LiNbO_3_ materials to explore the generation of a second harmonic through the incorporation of the BIC effect. Kim et al. [41] devised periodic arrays of LN nanodisks, enabling effective coupling of the conduction mode with the far field, resulting in the creation of a quasi-BIC structure. This inventive approach yielded a remarkable SHG conversion efficiency of 10^−2^ %. Similarly, Liu et al. [42] devised a two-layer LiNbO_3_ supersurface structure that harnessed both quasi-BIC modes and the chiral properties of the structure, achieving a conversion efficiency of 1.35 × 10^−2^ %. However, it is worth noting that many existing structural designs necessitate the etching of LN, leading to inherent drawbacks such as a low photodamage threshold and challenges associated with precise etching. Li et al. [43] designed a BIC-based semi-nonlinear photonic waveguide on an etch-free SiN/LNTF platform, which maximized the nonlinear mode overlap between FF and SH waves by mode phase matching, and achieved an experimental conversion efficiency as high as 4.05 % W^−1^ cm^−2^. When selecting substrate materials, researchers commonly opt for single-layer silica, with less consideration given to multilayer structures. Nevertheless, Qu et al. [44] recently achieved a second-harmonic conversion efficiency of 0.28 % at a light intensity of 6.3 GW/cm^2^ by employing a photonic crystal multilayer structure within a 1 µm thick LN film. The multilayer structure exhibits the capability to generate a photonic band gap, a crucial tool for manipulating and fine-tuning the properties of light propagation. The utilization of multilayer structures to generate BSWs confers inherent advantages over silica substrates. BSWs [45] represent electromagnetic waves that exist on the surface of periodic structures, showcasing distinctive propagation characteristics. By carefully adjusting the structural period and refractive index, the propagation properties of BSWs can be tailored to different wavelength ranges, facilitating local field enhancement. The confinement of energy near the surface, conferred by the propagation properties of BSWs, significantly amplifies the coupling strength, enabling the localization of the bound-state optical field within a continuous medium. This localization effect substantially augments the optical intensity and energy density of the bound state, thereby greatly enhancing the second-harmonic response of the structure. When grating waveguide structures are located on photonic crystals, the forbidden band effect further limits the range of light propagation, making the waveguide modes even more confined within the waveguide. In addition, photonic crystal structures typically have lower losses, and material selection and interlayer design can optimize the propagation path of light to significantly improve the quality factor. In contrast, silica substrates have high scattering losses over a range of frequencies. And photonic crystals can be integrated with other optical functional materials, such as quantum dots [46] and organic light-emitting materials [47]. This integration enables interactions with other optical materials.

In this investigation, we delve into the potential of enhancing the quasi-BIC effect in LN nanostructures through the implementation of BSWs supported by photonic crystal layers. The aim is to achieve efficient second-harmonic generation. To this end, we have made theoretical proposals and conducted corresponding simulation studies via finite element simulation. By manipulating the symmetry of the composite grating structure, we disrupt the perfect condition of BIC and successfully generate quasi-BIC states. To ensure optimal performance, we carefully select the materials for the grating and waveguide layers. Silicon dioxide is chosen as the grating layer material, while LN serves as the waveguide layer material. This selection mitigates issues such as surface quality damage and inhomogeneity that may arise from a direct etching of LN. Furthermore, it provides a higher refractive index contrast between the grating and the waveguide layer, effectively enhancing the confinement of the optical field within the LN. In addition, we opt for a photonic crystal substrate composed of alternating silicon nitride and silicon dioxide, rather than a silicon dioxide substrate. This choice amplifies the presence of BSWs, thereby augmenting the quasi-BIC effect. Strategic design of the photonic crystal structure and the properties of the continuum medium facilitate energy exchange and interactions. The all-dielectric nature enables excellent dispersion properties and avoids the ohmic loss. Our results demonstrate that the proposed structure achieves a remarkable second-harmonic conversion efficiency of up to 0.6 % (incident light intensity of 1.33 GW/cm^2^), with a LN film thickness of 200 nm. The innovative structure combines the unique advantages of BSW, BIC, LN, and all-dielectric materials. It not only enables flexible tailoring the structure dispersion to effectively suppress radiation loss but also facilitates strong coupling effects, leading to efficient second-harmonic generation. This research opens up promising avenues in various applications, including optical frequency converters, optical sensors, and integrated optical devices.

## 2 Structural design and optimization

**Figure 1:**
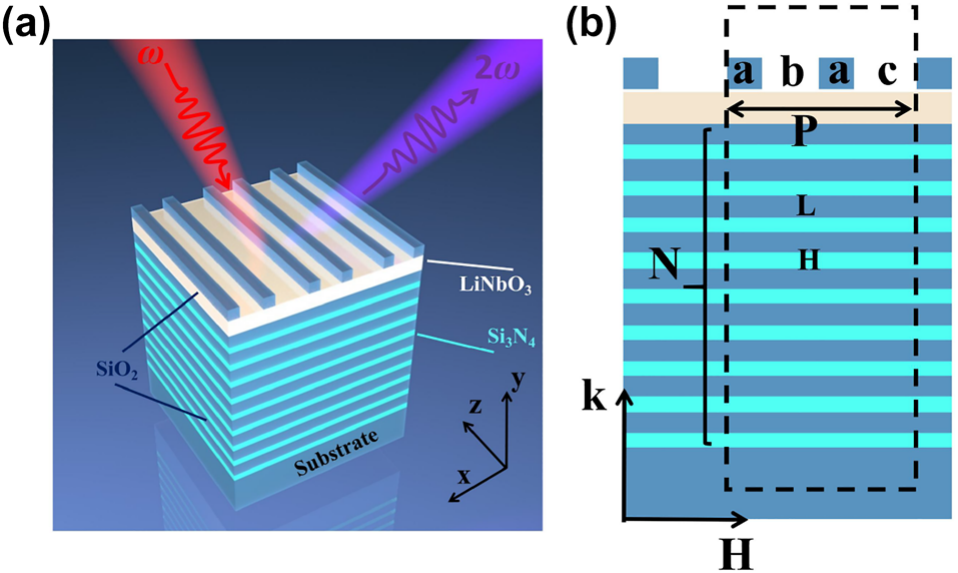
Schematic of a composite grating on a photonic crystal layer. (a) Three-dimensional schematic of a composite grating waveguide structure. (b) Two-dimensional schematic of the structure in the *x*–*y* plane, where the light is incident to the structure along the top of the grating and the TE polarized light is along the *z*-direction.

The three-dimensional schematic of the proposed structure is presented in Figure 1(a). The photonic crystal layers comprise alternating Si_3_N_4_ and SiO_2_ materials, exhibiting negligible loss within the considered wavelength range. The LN waveguide layer is situated between the photonic crystal and the grating layer, with the SiO_2_ grating layer positioned at the top. The grating layer introduces an additional wave vector, while the waveguide layer supports the guided mode. The DBR layer facilitates weak coupling between the BSW and Fano modes. In Figure 1(b), the two-dimensional parameters of the structure are characterized. The high and low refractive index materials within the photonic crystal layer are denoted by *H* and *L*, respectively, with the logarithm represented as *N*. The composite grating exhibits a periodicity referred to as P, where “a” denotes the width of the grating, and “b” and “c” represent the widths of the unetched regions of the grating. The thickness of the grating and the LiNbO_3_ film are denoted as “*h*_1_” and “*h*_2_,” respectively. This configuration defines an asymmetric geometric factor $\delta =\frac{b-c}{b+c}$ , which satisfies $\delta \in \left[-1,1\right]$ . As cited in [48], LN exhibits anisotropic properties. The refractive index of SiO_2_ is obtained from [49], while Si_3_N_4_’s refractive index is derived from [50]. When considering a TE polarized incident from the top of the grating, the phase-matching condition that must be met when *δ* ≠ 0 can be expressed as follows:

(1) $$k_{\mathrm{BSW}}=-k_{0}n_{0}\sin\theta +\frac{2m\pi }{P}$$

with *k*_*BSW*_ being the BSW wave vector, $k_{0}=\frac{2\pi }{\lambda }$ is the vacuum wave vector, *λ* represents the wavelength of the incident light in vacuum, and *θ* is the incident angle. For the purposes of this investigation, we assume a refractive index *n*_0_ = 1 for the surrounding medium. When *δ* = 0, the period of the grating, denoted as *P*, is reduced to *P*_1_, where $P_{1}=\frac{P}{2}$ . The phase matching condition becomes:

(2) $$k_{\mathrm{BSW}}=-k_{0}n_{0}\sin\theta +\frac{4m\pi }{P}$$

**Figure 2:**
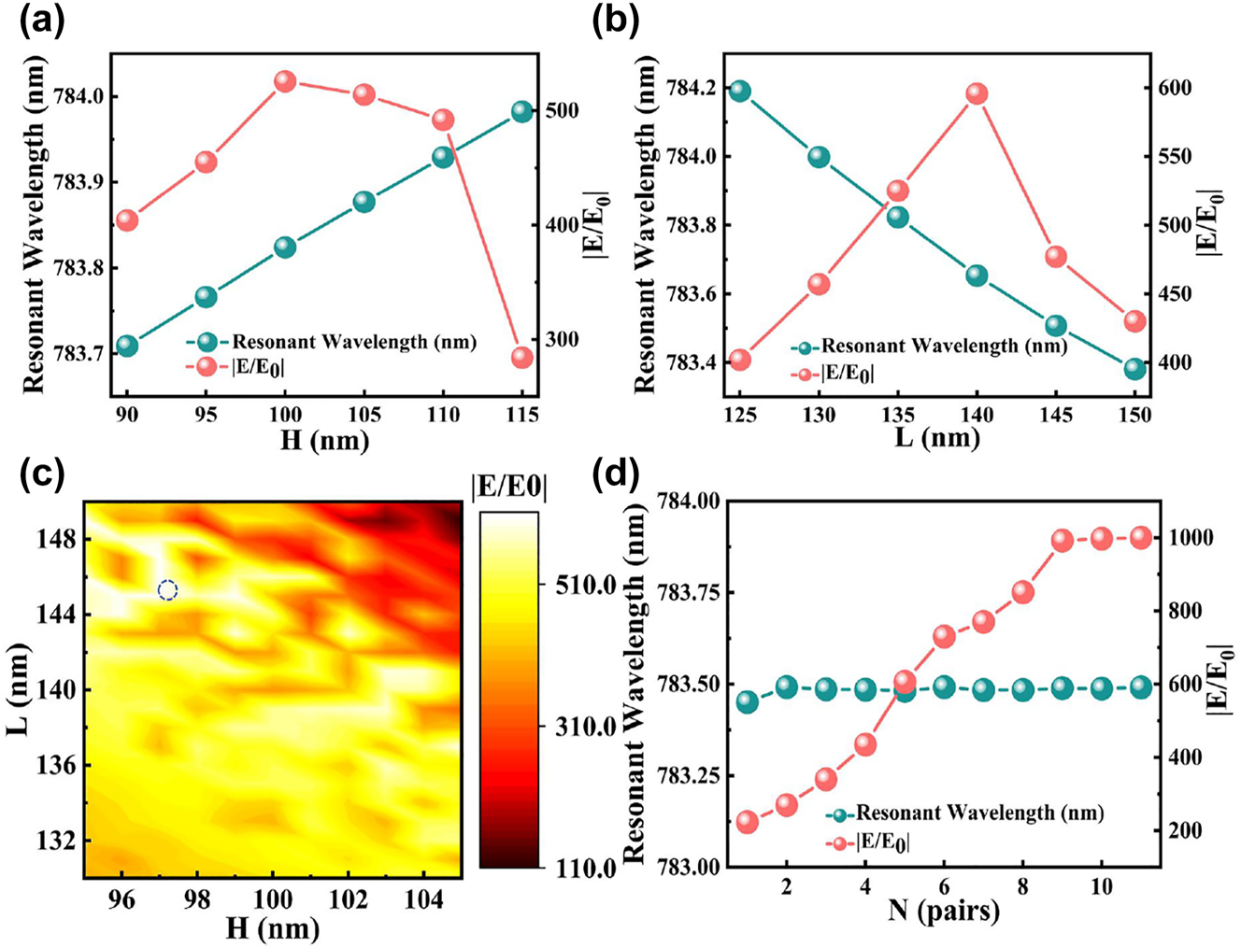
Optimization of photonic crystal layers. (a) Resonant wavelength and electric field enhancement corresponding to changing the thickness *H* of the photonic crystal layer. (b) Resonant wavelength and electric field enhancement correspond to changing the thickness *L* of the photonic crystal layer. (c) Fine optimization of the relationship between the electric field intensity values near *H* = 100 nm, *L* = 140 nm. (d) Relationship between the number of pairs of photonic crystal layers and the value of electric field enhancement and resonance wavelength for the considered parameter values.

Maximizing the Bloch surface wave effect necessitates the optimization of the thickness parameters of the photonic crystal layer. Following the Bragg condition [51], wherein $n_{1}L\approx n_{2}H\approx \frac{\lambda }{4}$ , where *n*_1_ and *n*_2_ represent the refractive indices of the two dielectric materials respectively, and *λ* denotes the resonant wavelength, the optimization process is conducted within the region satisfying the Bragg condition. Assuming the values *P* = 400 nm, *a* = 80 nm, *δ* = −0.1, *h*_1_ = 80 nm, *h*_2_ = 200 nm, and *N* = 5. The first step involves fixing *L* = 140 nm and observing the changes in resonance wavelength and local field strength as *H* is varied, as depicted in Figure 2(a). With an increase in *H*, the resonance wavelength experiences a monotonic redshift, while the local field strength reaches its peak at *H* = 100 nm. Subsequently, with *H* held constant at 100 nm, the variation in the resonance wavelength as *L* is changed is illustrated in Figure 2(b). As increases, a blue-shifted trend in the resonance wavelength is observed, with the highest electric field strength being visible at *L* = 140 nm. Furthermore, a more refined optimization around *H* = 100 nm and *L* = 140 nm is demonstrated in Figure 2(c). The optimal local field intensity is achieved at *H* = 97 nm and *L* = 145 nm, as indicated by the blue dashed circle in Figure 2(c).

The variation in local field strength is influenced by the quantity of photonic crystal layer pairs in a given configuration. In this study, we consider the assumptions of *P* = 400 nm, *a* = 80 nm, *δ* = −0.1, *H* = 97 nm, and *L* = 145 nm. Figure 2(d) illustrates the investigation into the relationship between the number of pairs of different photonic crystals and the local field strength. As the number of pairs increases, the electric field intensity experiences a corresponding rise. This behavior can be attributed to the reduction in radiation loss. However, it is worth noting that the field enhancement does not tend toward infinity as the number of pairs continues to increase. This is due to the inherent inability to completely eliminate losses. The selection of *N* = 9 as the logarithm for the photonic crystal layer is based on the observation that optimal outcomes were consistently attained within the range under consideration.

## 3 Theoretical analysis

**Figure 3:**
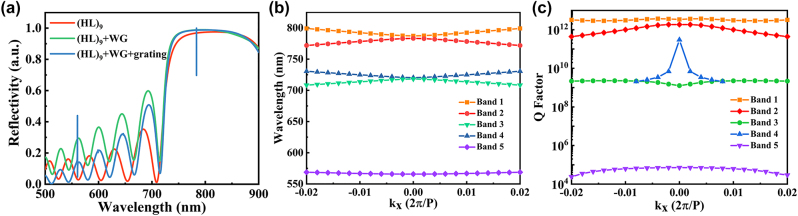
Analysis of structural resonance mechanisms. (a) The reflectance spectra corresponding to different wavelengths under TE mode normal incidence. The black line indicates the structure with the photonic crystal layer only, the red line indicates the addition of the waveguide layer, and the blue line indicates the addition of the grating waveguide layer. (b) Dispersion relation diagram of the proposed structure in the first Brillouin zone. (c) *Q* factor from bands 1 to 5 in the first Brillouin zone.

To assess the structural response to the incident wave, we investigated three scenarios: (1) only the photonic crystal layer, (2) incorporating the waveguide layer, and (3) introducing the grating waveguide layer. The geometric parameters of the structure remained consistent with the aforementioned study. Figure 3(a) presents the corresponding reflection spectra. It is noteworthy that the photonic crystal layer exhibits a forbidden band within the wavelength range of 700 nm–900 nm. This phenomenon arises due to the destructive interference of reflected waves at the interfaces of the SiO_2_ and Si_3_N_4_ materials in the DBR. Consequently, light is unable to propagate through the structure during this band. Upon the inclusion of the waveguide layer, a dispersion relationship emerges below the light cone in the air medium. As a result, the incident light fails to couple with the waveguide, rendering transmission unfeasible even with the waveguide present. However, upon further incorporating the grating layer, an alteration becomes apparent. A splitting effect occurs near the original forbidden band at approximately 783.5 nm, resulting in an exceedingly narrow Fano resonance peak. This occurrence stems from the interplay of the DBR’s reflection and the cavity resonance, which facilitates the output of the optical field at this specific position. Consequently, the spectrum exhibits an asymmetric tilt due to the interference between two distinct modes. The field intensity undergoes a substantial change at this specific position, implying that the resonance owes its existence to the phase-matching conditions provided by the coupled grating.

The eigenfrequency module implemented in Comsol Multiphysics is utilized to obtain solutions for the structural eigenvalues. The simulation employs a periodic boundary condition along the *x*-direction, while a perfectly matched layer is employed along the *y*-direction. The outcomes of the computations are presented in Figure 3(b). Within the wavelength range of 550 nm–850 nm, five energy bands manifest, corresponding to five distinct eigenmodes. The dispersion of these energy bands is examined in terms of the forward and backward wave dispersion relations, characterized by the curves at *k*_*x*_ < 0 and *k*_*x*_ > 0, respectively. It is worth mentioning that bands 1 to 4 exhibit linear dispersion, while band 5 demonstrates curved dispersion, as deduced from the features exhibited by the energy bands. The *Q* factors corresponding to the five energy bands are shown in Figure 3(c).

In the spectral range encompassing bands 1 to 4, the spatial profiles of the electric and magnetic fields associated with the specific location Γ (wavevector *k*_*x*_ = 0) are extracted. A manifestation of a photonic crystal bandgap’s partial discontinuity is the emergence of a resonant peak, an occurrence observed when the grating’s periodicity is smaller than the operational wavelength [52]. The extent of this opening is contingent upon the strength of the coupling between the forward and backward waves. It is worth noting, as depicted in Figure 3(b) at point Γ, that bands 1 and 2 exhibit nondegeneracy, while bands 3 and 4 also display nondegeneracy [53].

**Figure 4:**
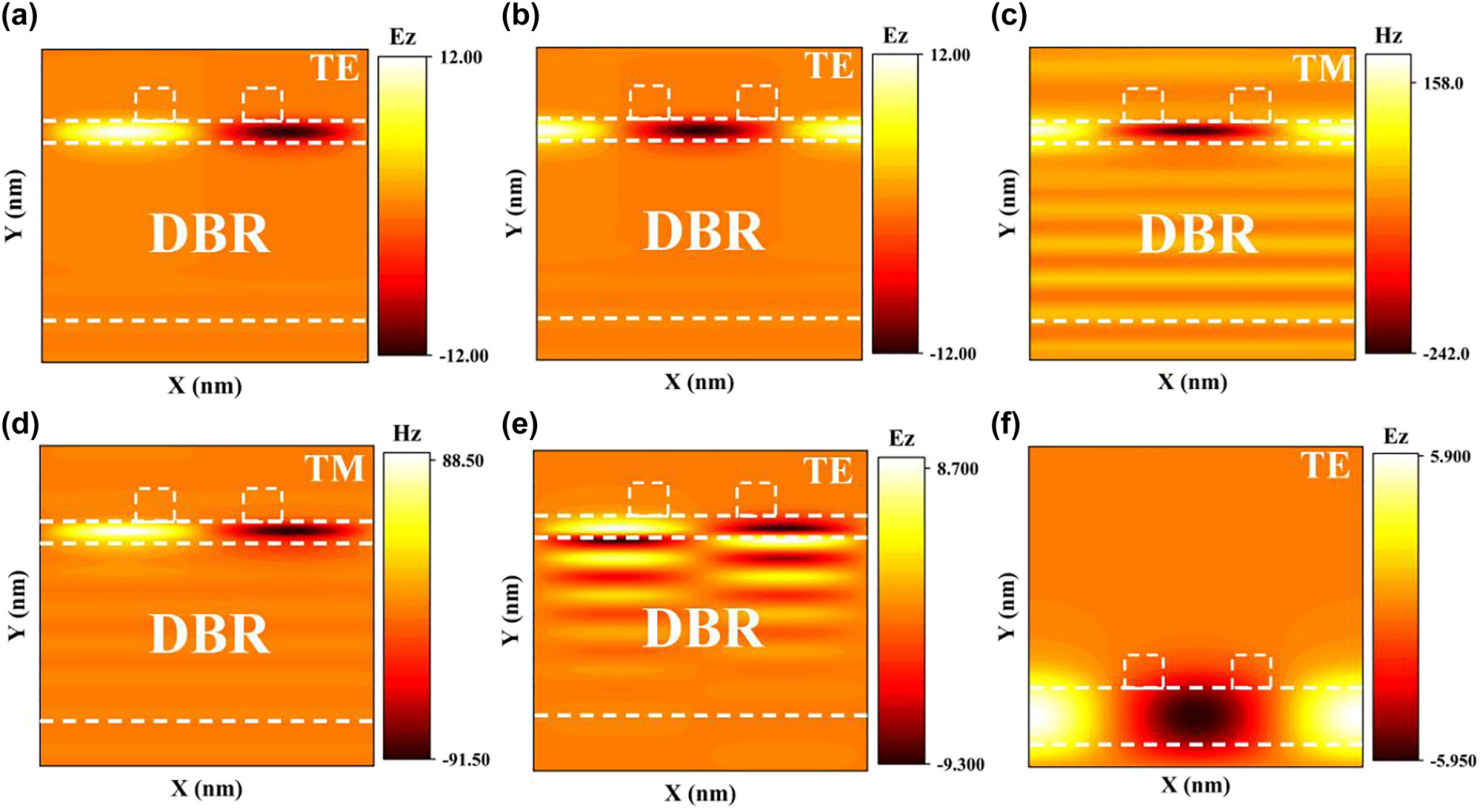
Bands corresponding electric or magnetic field diagrams. (a–e) The electric or magnetic field distribution at position *k*_*x*_ = 0 corresponds to bands 1 to 5 in Figure 3(b), respectively. (f) Plot of the electric field at the position corresponding to *k*_*x*_ = 0 for the case of grating waveguide.

In Figure 4(a–d), the distribution of electric or magnetic fields for bands 1–4 is depicted, where bands 1–2 represent the TE mode, and bands 3–4 represent the TM mode. These findings indicate that the proposed structure possesses the capability to support both TE and TM modes. Notably, bands 1 and 4 exhibit an odd symmetry with respect to *x* = 0, while bands 2 and 3 display an even symmetry about the same axis. Building upon the principles governing symmetry-protected and symmetry-unprotected BICs [35], it is observed that the odd BIC remains largely unaffected by structural symmetry, whereas the even BIC is influenced by it. In the TE mode, the even BIC experiences modulation due to geometric asymmetry, leading to the formation of a leaky mode. This phenomenon is responsible for the emergence of a distinct resonance peak near 783.5 nm in Figure 3(a), corresponding to band 2. Conversely, band 1 does not display a resonance peak but rather assumes the characteristics of a dark mode in Figure 3. This behavior can be attributed to the inability of the incident wave to couple with this mode, allowing it to persist in the BIC state.

As depicted in Figure 4(b), the distribution of band 2 primarily localizes within the LN waveguide layer. However, solely relying on the waveguide layer fails to elicit the occurrence of the bound states in the continuum. To investigate the origin of the symmetric BIC within the proposed structure, the mode is examined in the scenario where only the grating waveguide is present, as shown in Figure 4(f). It can be observed that the electric field distribution diagram, facilitated by the grating, exhibits similarities to that of Figure 4(b). Nonetheless, the electric field intensity is comparatively reduced compared to the configuration incorporating the distributed Bragg reflector. This observation suggests that the grating layer furnishes the necessary prerequisites for the generation of resonance, which collectively contributes to the generation of BIC under the influence of the BSW satisfying the Bragg condition.

The electric field profile associated with the Γ point in band 5 is depicted in Figure 4(e), demonstrating a notable concentration of the field within the waveguide layer. The field exhibits an exponential decay along the distributed Bragg reflector layer and a rapid decay along the air layer, as is typical of BSWs [54]–[56]. This behavior stems from the dispersion folding of BSWs in the DBR, induced by the lateral periodicity of the grating. The resonant peak observed at approximately 565 nm in Figure 3(a) corresponds to the so-called BSW mode. While several additional resonant peaks are discernible in the reflection spectrum displayed in Figure 3(a), they do not appear in the dispersion relation diagram. These peaks represent the leakage mode arising from the interaction between the grating waveguide and the photonic crystal layer. These modes are treated as pseudo modes, so they are not considered in the energy band computation.

Several structures are formed by composite resonances, which cannot be discerned solely from the diagram depicting the distribution of the electric field. In order to gain a clearer understanding of the generation mechanism, the utilization of multipole analysis becomes imperative to accurately ascertain the interactions between different polarons. Decomposition of the multipole in a Cartesian coordinate system facilitates the acquisition of the multipole moment through initial integration of the electric field distribution, subsequently enabling the computation of the scattering power in the far-field. The equation for the polar moment is provided in accordance with the reference cited as Ref. [57].

**Figure 5:**
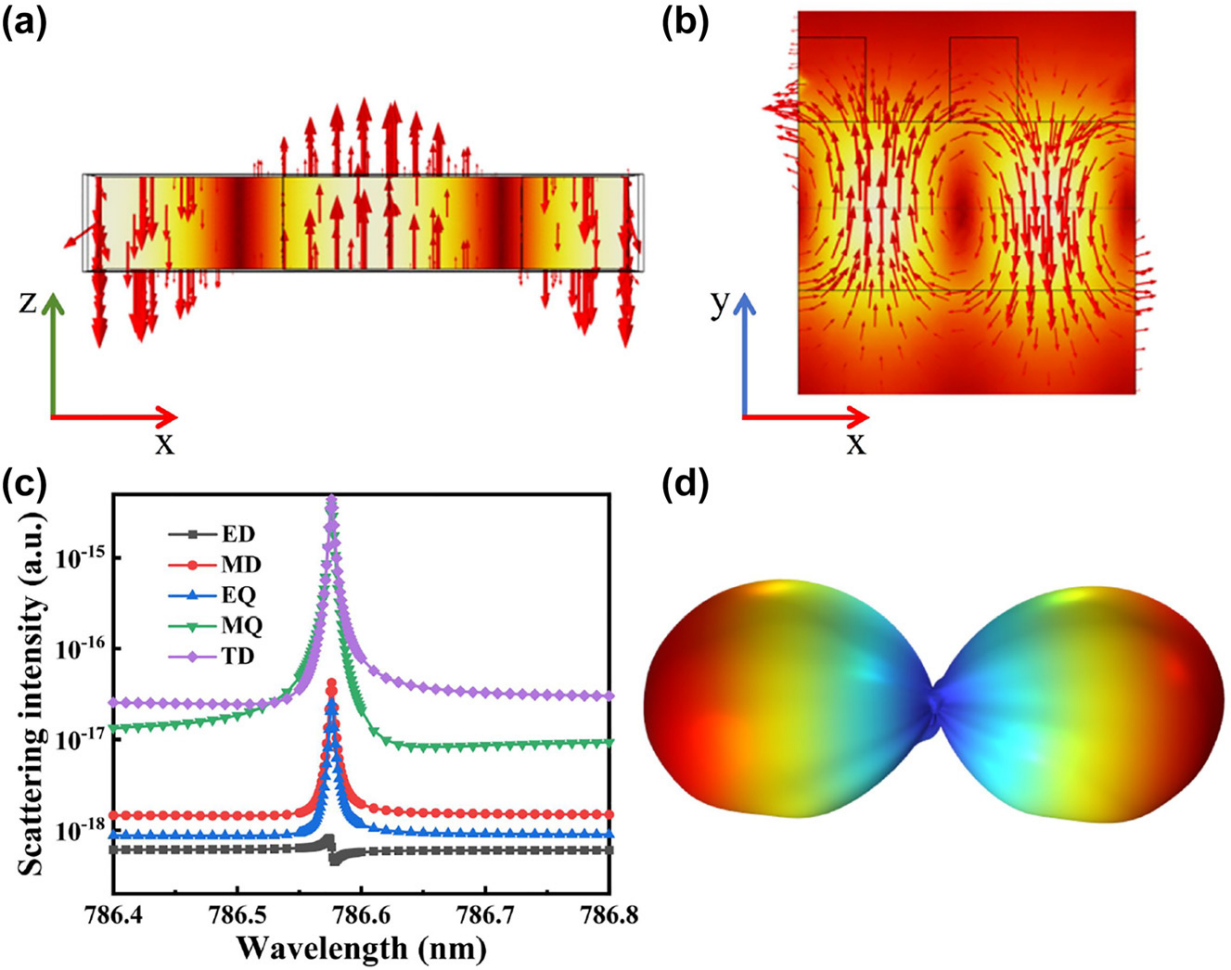
Multipole analysis. (a) The electric field distribution at the resonant wavelength with arrows indicating the displacement current direction. (b) The magnetic field distribution at the resonant wavelength with arrows indicating the direction of the displacement current. (c) The scattered power distribution of various multipoles near the resonant wavelength. (d) The far-field diagram at the resonant wavelength.

Our proposed structure can be conceptualized as a meta-structure characterized by a duty cycle of 1 in the *z*-direction. The distribution diagrams depicting the electric and magnetic fields are presented in Figure 5(a) and (b), respectively. These diagrams are accompanied by arrows indicating the direction of current flow. Figure 5(c) showcases the individual contributions of each multipole, which have been computed using the multipole formula. The geometric parameters employed in this calculation are consistent with those utilized in Figure 3(a). The level of refinement in the 3D grid impacts the slight wavelength shift observed. From a quantitative perspective, it is evident that the dominant mode at the resonance wavelength is the toroid dipole (TD) mode, thus confirming it as a TD resonance. The intensity for this resonance is approximately two times higher compared to the magnetic quadrupole (MQ) mode, around two orders of magnitude higher than the magnetic dipole (MD) and electric quadrupole (EQ) modes, and approximately three orders of magnitude higher than the electric dipole (ED) mode. Additionally, the energies of the TD and MQ modes coincide nearly at the resonant position, leading to an interference-induced phase extinction and cancellation of the outgoing wave. This phenomenon elucidates the manifestation of the high *Q* value. The far-field diagram presented in Figure 5(d) exhibits a slight defect due to field leakage. This distinctive far-field pattern corresponds to a typical manifestation of the TD mode, thus further substantiating the accuracy of our meticulous calculations.

## 4 Results and discussions

### 4.1 Linear simulation and calculation

**Figure 6:**
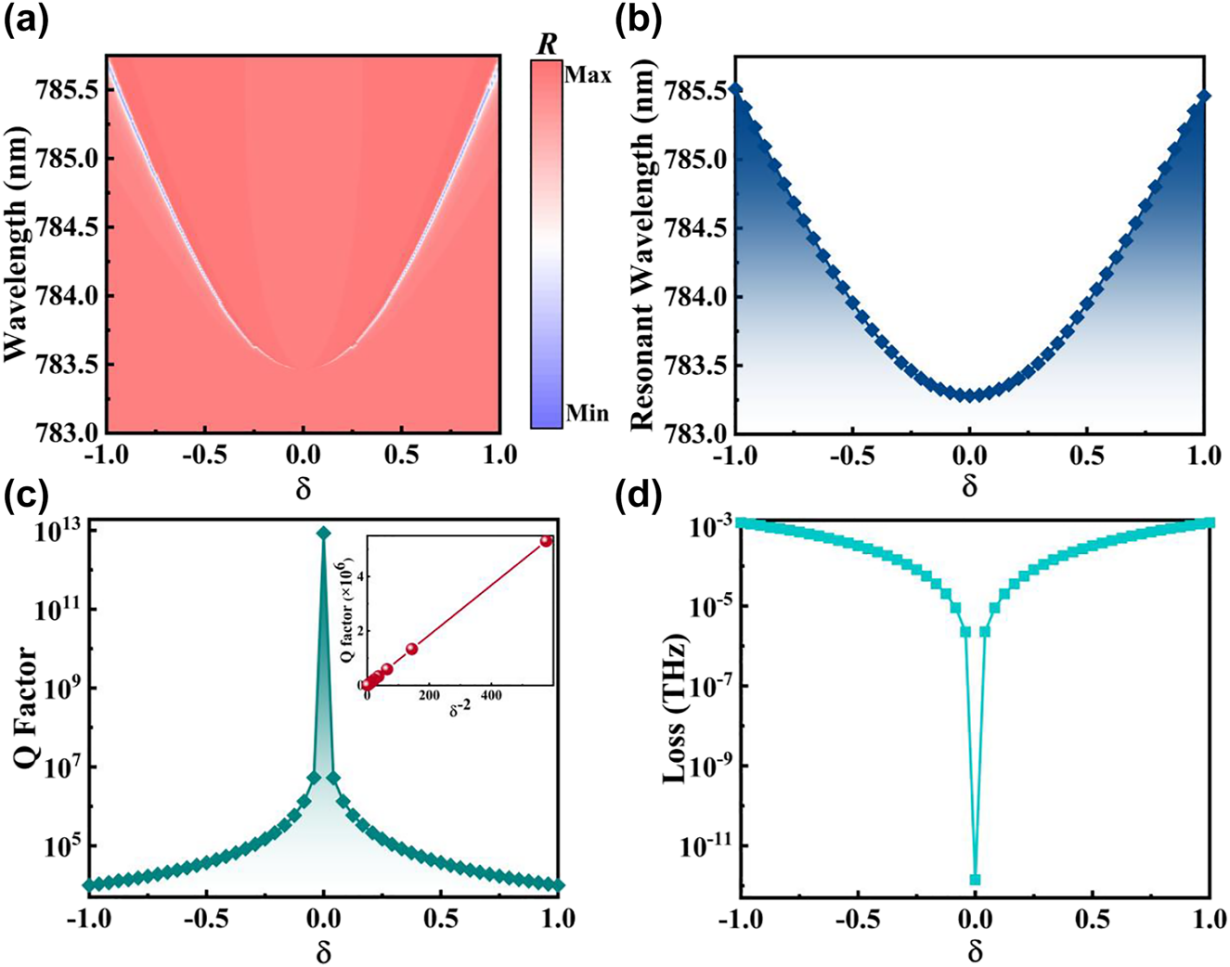
Reflectance spectra correspond to energy band calculations. (a) Relation between reflection spectrum and wavelength for $\delta \in \left[-1,1\right]$ . (b) Dispersion relation between the calculated resonant wavelength and *δ* for $\delta \in \left[-1,1\right]$ . (c) The corresponding *Q*-factor value when varying *δ*. The inset shows that *Q* is positively correlated with *δ* ^−2^. (d) The corresponding loss value when varying *δ*.

Modulating the Fano line by breaking the structural geometric asymmetry has been established as a well-established approach [58], [59] for achieving the quasi-BIC phenomenon. Figure 6(a) illustrates the reflection spectrum obtained by varying the parameter *δ* within the range of [−1, 1] while keeping other conditions constant. Notably, when *δ* is set to 0, the line width disappears due to the radiated wave’s vanishing coupling constant, resulting in an even BIC’s emergence. However, as the absolute value of *δ* gradually approaches 1, the spectral resonance linewidth broadens and the BIC transitions into a quasi-BIC. This observation implies that the stability of the even BIC is influenced by the presence of geometric asymmetry, corroborating previous theoretical findings.

Owing to the consistent symmetry exhibited by the grating distribution at equal absolute values of *δ*, it follows that the reflection spectrum displays a symmetric distribution around *δ* = 0. The resonant wavelength manifests minor fluctuations within a confined range while *δ* varies between −1 and 1. This slight variation arises from the subtle modification in the equivalent refractive index, owing to the displacement of the grating block. As depicted in Figure 6(b), we have computed the dispersion relation by systematically varying *δ*, and the resulting reflectance spectrum is largely consistent with the observed data. To ascertain the *Q*-factor, we adopt the equation $Q=\frac{\omega_{0}}{2\gamma }$ , with *ω*_0_ denoting the resonant frequency and *γ* representing the damping rate. Figure 6(c) reveals a pronounced response in the *Q* factor as *δ* varies, with the *Q* value undergoing a seven-order-of-magnitude change during the movement of *δ* between the interval [−1, 1]. The inset in the figure illustrates the relationship between the *Q* factor and *δ* ^−2^. The fitting results confirm that the *Q* factor can be represented by a linear function of the inverse of the square of the asymmetric parameter, which aligns with the proposed perturbation theory [60]. The associated losses, depicted in Figure 6(d), escalate as the absolute value of *δ* increases.

**Figure 7:**
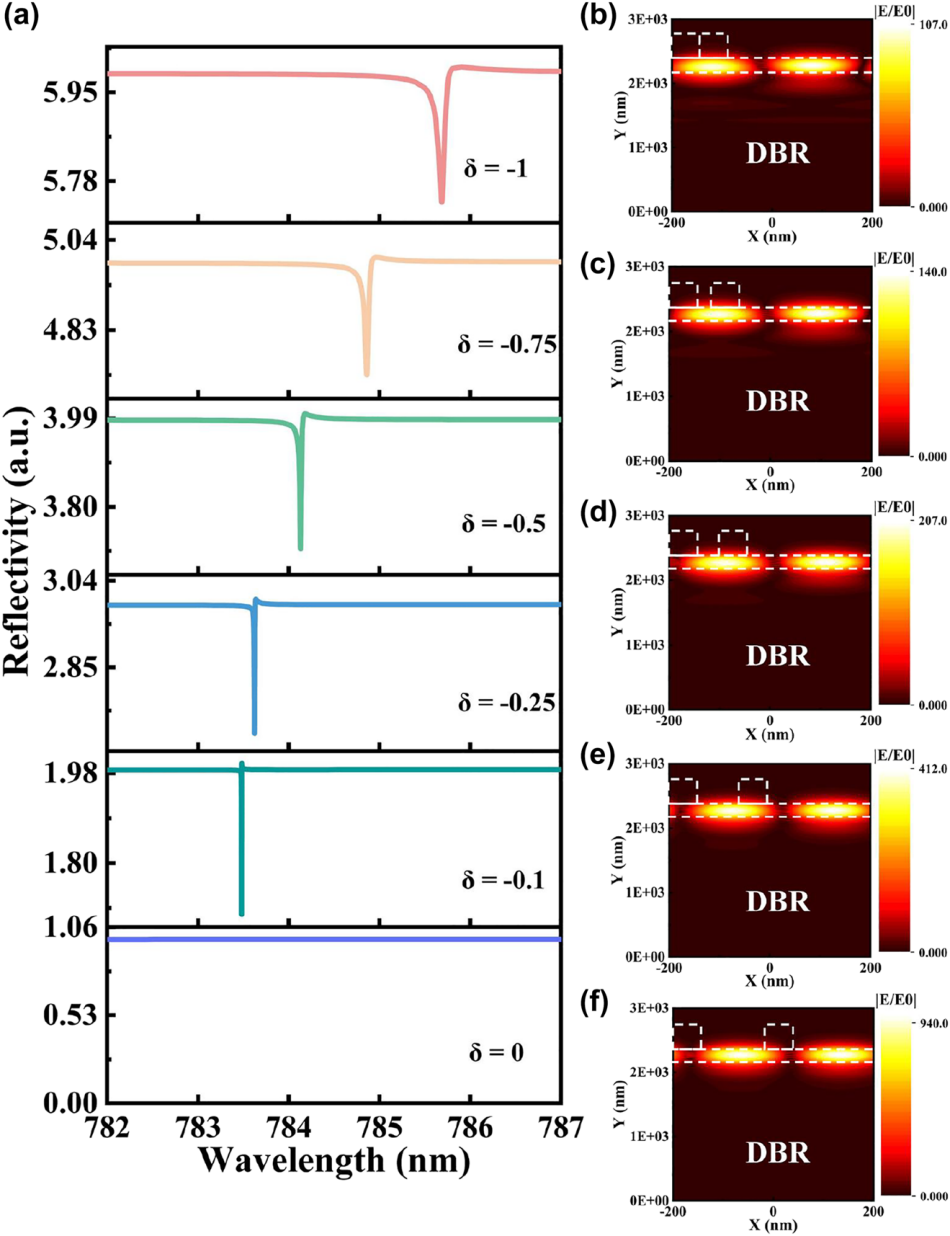
Plot of electric field distribution at different asymmetry factors. (a) Relationship between the reflected spectrum and the wavelength for varying *δ*. (b–f) The corresponding electric field distribution at the resonant wavelengths for *δ* = −1, −0.75, −0.5, −0.25, and −0.1.

The reflection spectra in TE mode are computed by varying the parameter *δ* within the range of −1 to 0. Figure 7(a) visually demonstrates that the Fano resonance peak gradually becomes narrower as the magnitude of *δ* decreases, eventually vanishing at *δ* = 0. This behavior arises due to the absence of coupling between the incident wave and the mode. The subsequent Figure 7(b–f) offer insight into the corresponding distribution of the electric field for different values of *δ* (−1, −0.75, −0.5, −0.25, and −0.1). Notably, for *δ* > 0, the electric field pattern concurs with the aforementioned results for *δ* < 0; however, it is omitted herein for brevity. The graphical representation reveals that as *δ* approaches −1, there is a discernible increase in the extent of leakage toward the substrate. Conversely, a reduction in the magnitude of absolute value *δ* leads to a decrease in substrate leakage. Consequently, a more tightly confined normalized electric field is achieved within the waveguide layer, presenting a novel strategy for amplifying the interaction between light and matter.

**Figure 8:**
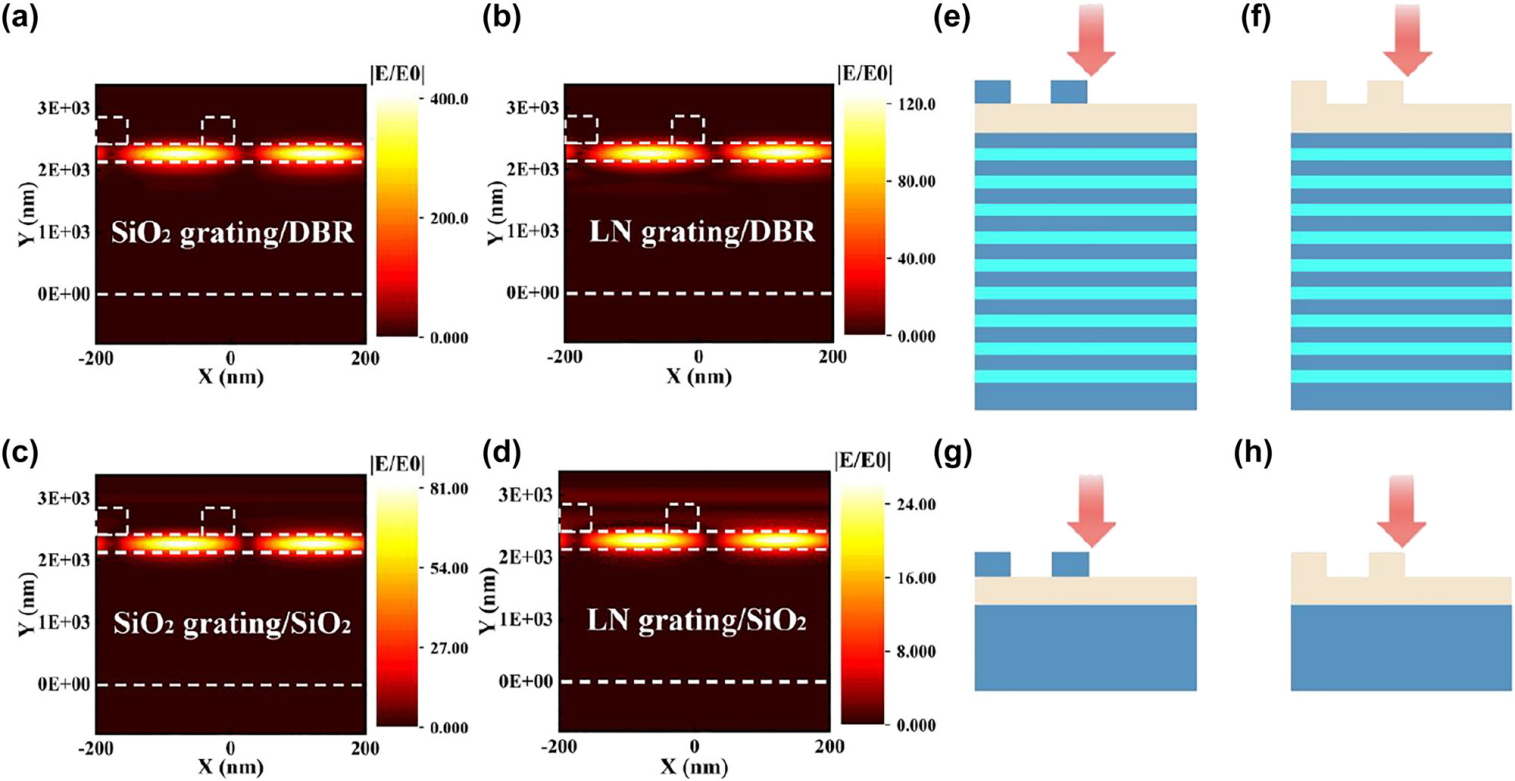
Comparison of electric field enhancement values for different structures. (a–d) The electric field distribution of the four structures corresponding to the change of grating material and substrate parameters, respectively. (e–h) Two-dimensional plane diagrams corresponding to the electric field figures in (a–d).

In this study, we present a comparative analysis of the results obtained through variations in the grating layer material and a comparison between the photonic crystal and SiO_2_ substrate, as illustrated in Figure 8. By maintaining consistent values for other parameters and setting *δ* to −0.25, it becomes apparent from the comparison between Figure 8(a) and (b) that SiO_2_, when employed as the grating material, achieves a threefold increase in local field enhancement compared to LiNbO_3_. This enhancement arises from the substantial contrast in refractive indices between the grating and the waveguide layer, which facilitates superior confinement of the mode within the waveguide layer. Additionally, the loss-free characteristics of SiO_2_ at the resonant wavelength also contribute to the amplification of field strength. Figure 8(a) further portrays a similar fivefold enhancement in the local field when employing a DBR as the substrate, in comparison to the SiO_2_ substrate depicted in Figure 8(c). This notable enhancement is attributed to the increased structural flexibility offered by the multilayer configuration, along with the capacity of the DBR to mitigate diffraction toward the substrate layer. Consequently, this arrangement achieves a more pronounced effect in terms of localized field strength, while satisfying the Bragg condition. Moreover, when utilizing an LN grating with the SiO_2_ substrate, the field limiting capability attained under identical conditions is an order of magnitude lower than that achieved by the constructed structure. This significant difference further underscores the superiority of our designed configuration in enhancing field effects. The schematic representations of the two-dimensional planar structures corresponding to Figure 8(a–d) are presented in Figure 8(e–h).

### 4.2 Nonlinear simulation and calculation

**Figure 9:**
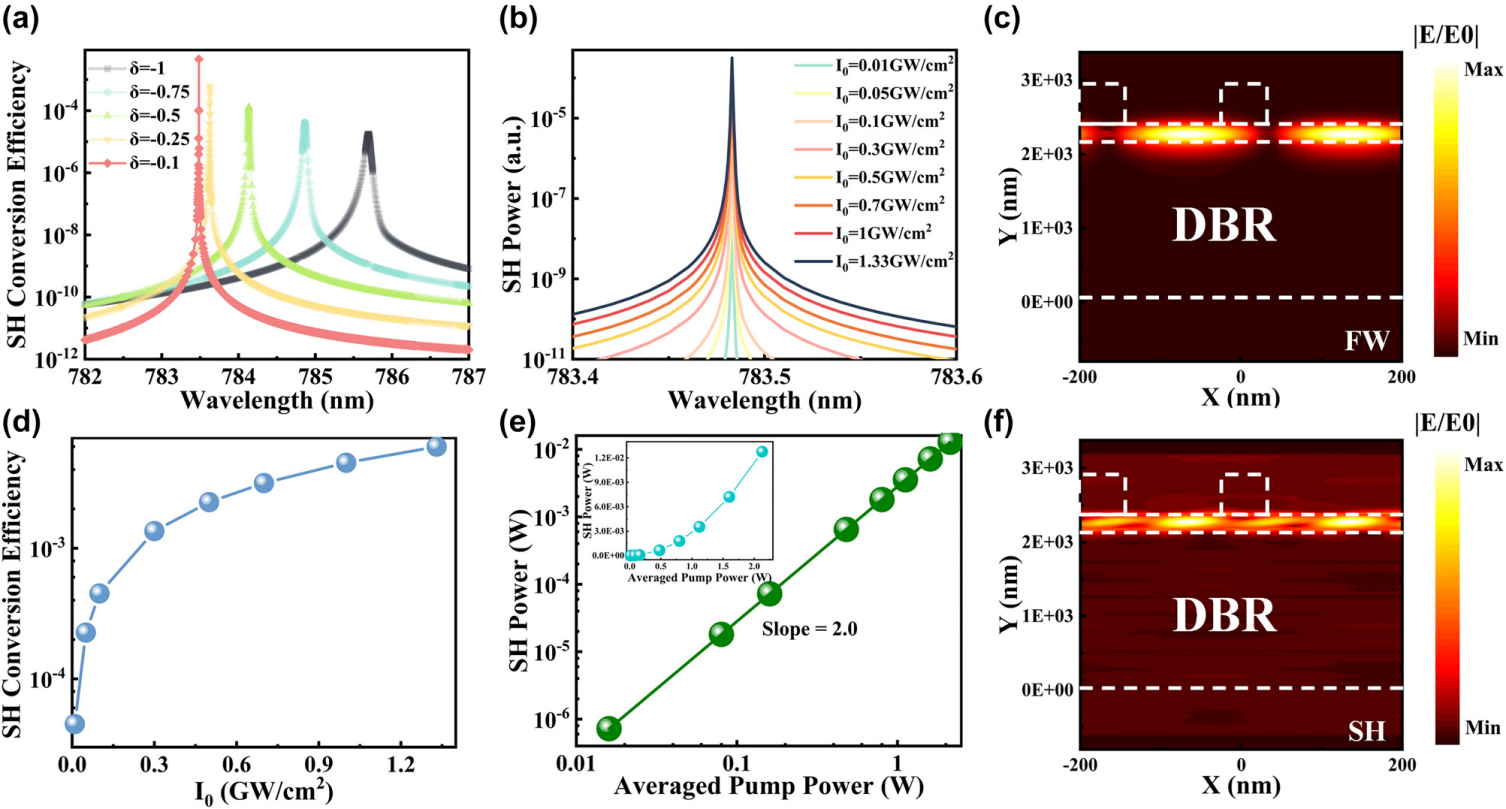
Nonlinear simulation. (a) The relationship between the SH conversion efficiency and the wavelength at different asymmetry factor. (b) The relationship between the SH power and wavelength at different light intensities. (c) The distribution of the electric field at the fundamental frequency. (d) The relationship between the SH conversion efficiency and light intensity at resonant wavelength. (e) The relationship between the SH power and the averaged pump power at resonant wavelengths, and the inset shows the original data when no logarithm is taken. (f) The distribution of the electric field at the doubling frequency.

Utilizing the finely tuned structural parameters elucidated earlier, we conduct simulations employing the nonlinear solver at a designated value of *δ* = −0.1. The second-order nonlinear polarization tensor of LN [61] is mathematically represented as follows:

(3) $$\begin{array}{ll} \left(\begin{array}{c} P_{x}^{NL}\\ P_{y}^{NL}\\ P_{z}^{NL} \end{array}\right) & =2\varepsilon_{0}\left(\begin{array}{cccccc} d_{33} & d_{31} & d_{31} & 0 & 0 & 0\\ 0 & d_{22} & -d_{22} & 0 & 0 & d_{31}\\ 0 & 0 & -d_{22} & d_{31} & 0 & 0 \end{array}\right)\left(\begin{array}{c} E_{x}^{2}\\ E_{y}^{2}\\ E_{z}^{2}\\ 2E_{z}E_{y}\\ 2E_{z}E_{x}\\ 2E_{x}E_{y} \end{array}\right) \end{array}$$

where *d*_33_ = 25.2 pm/V, *d*_31_ = 4.1 pm/V, and *d*_22_ = 2.1 pm/V. The conversion efficiency of the second harmonic can be determined using the following equation [62]:

(4) $$\eta_{SH}=\frac{P_{SH}}{P_{FW}}$$

where *P*_*SH*_ denotes the second-harmonic power and *P*_*FW*_ denotes the fundamental wave power. The relationship between the incident wavelength and the second-harmonic conversion efficiency obtained by varying the asymmetry factor under positive light incidence is illustrated in Figure 9(a). It can be observed that as the absolute value *δ* decreases, the corresponding conversion efficiency increases. The correlation between variations in light intensity at the resonant wavelength and the SH power can be observed in Figure 9(b). The resonant wavelength corresponds to the maximum power. The conversion efficiency exhibits a synchronized increase with the intensity of light, up to a value of 4.5 × 10^−3^ at 1 GW/cm^2^ in Figure 9(d). Figure 9(e), where the relationship between averaged pump power and SH power is demonstrated. The inset within the figure provides the original dataset for reference purposes. By taking the logarithm of both the horizontal and vertical axes, a slope of two can be derived, thereby confirming adherence to a second-order nonlinear relationship. Figure 9(c and f) depicts the corresponding fundamental frequency and doubling electric field diagrams.

**Figure 10:**
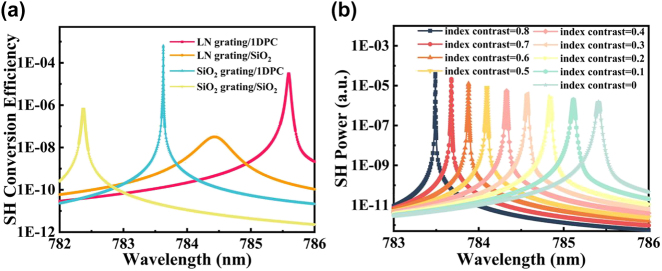
Comparison of SHG of structures. (a) Relationship between resonant wavelength and SH conversion efficiency for the designed structure and the three contrasting structures at 1 GW/cm^2^ light intensity. (b) The relationship between the SH power and wavelength at the respective resonant wavelengths for different refractive index contrasts.

Figure 10(a) compares the SH conversion efficiency exhibited by the four structures illustrated in Figure 8, considering their respective resonant wavelengths. The observations indicate a positive correlation between the strength of the local field at the fundamental frequency and the magnitude of the conversion efficiency achieved. The proposed structure improves by one order of magnitude compared to using LN as the grating material, three orders of magnitude compared to using SiO_2_ as the substrate, and four orders of magnitude compared to using LN as the grating material and using SiO_2_ as the substrate. To investigate this further, we introduce the concept of refractive index contrast, defined as the difference between a value of 2.2 and the refractive index of the grating under examination. Figure 10(b) demonstrates the analysis of the SH power trend across different refractive index contrasts. It becomes apparent that a higher refractive index contrast leads to sharper peaks and narrower bandwidths, resulting in a greater SH power magnitude.

In Table 1, we present an overview of the correlation between the local field strength values and the conversion efficiency for the construction employing LiNbO_3_ as a nonlinear material. Drawing upon the aforementioned discoveries, it becomes evident that our proposed architecture holds the potential to attain conversion efficiencies on the magnitude of 10^−3^, surpassing the previously accessible data by a minimum of one order of magnitude. Notably, the proximity of the conversion efficiency to the BIC enables further enhancements in its performance.

**Table 1:** Comparison of SHG conversion efficiencies generated by different structures of LiNbO_3_ as a nonlinear material.

Structure [Ref.]	Fundamental wavelength	Pump intensity $\left(I_{FW}^{\mathrm{peak}}\right)$	|*E*/*E*_0_|	*η*_*SH*_	$\eta_{norm}=\frac{\eta_{SH}}{I_{FW}^{\mathrm{peak}}}\;\left(\text{c}{\text{m}}^{2}/\text{GW}\right)$
LiNbO_3_ photonic crystal L_3_ cavity [63]	1390 nm	53 μW	–	6.4 × 10^−9^	–
LiNbO_3_ on Al substrate [64]	351.3 nm	5.31 GW/cm^2^	22	1.1528 × 10^−5^	2.1 × 10^−6^
LGNW on SiO_2_ substrate [33]	690 nm	1.33 GW/cm^2^	35	8.13 × 10^−5^	6.1 × 10^−5^
LiNbO_3_ on HMM [65]	565.4 nm	5.31 GW/cm^2^	35	5.1371 × 10^−5^	9.6 × 10^−6^
LiNbO_3_ metasurface [66]	800 nm	2.05 GW/cm^2^	–	2 × 10^−6^	9.7 × 10^−7^
Monolithic LiNbO_3_ metasurface [67]	830 nm	0.5 GW/cm^2^	30	2.4 × 10^−8^	4.8 × 10^−8^
Nanostructured LiNbO_3_ [68]	1605 nm	3.2 GW/cm^2^	36	3.165 × 10^−4^	9.8 × 10^−5^
LN RWG nanostructure [69]	1549.81 nm	–	–	1 × 10^−4^	–
**LNGW on 1DPC platform [this work]**	**783.5 nm**	**1.33 GW/cm** ^**2**^	**940**	**6** × **10** ^**−3**^	**4.5** × **10** ^**−3**^

## 5 Conclusions

In conclusion, we have designed a composite grating waveguide structure supporting Bloch surface wave to introduce asymmetry in the grating spacing. By harnessing the powerful BSW, we have successfully enhanced the transition from bound to quasi-BIC states in the continuum. This proposed configuration, characterized by its compact size and efficient nonlinear conversion, exhibits an impressive *Q* factor of 10^6^. To shed light on the origins of the high *Q* factor, we have calculated the energy bands and performed a multipole analysis. Through structure optimization, we have achieved a remarkable normalized electric field strength value of 940 in the TE mode, with an asymmetry factor of −0.1 of the composite gratings. Notably, our proposed structure demonstrates an outstanding conversion efficiency of 6 × 10^−3^ and a normalized conversion efficiency of 4.5 × 10^−3^. These figures surpass previous structures by at least one order of magnitude. The utilization of photonic crystal layer structures in micro- and nano-sized designs represents a promising avenue for the advancement of next-generation optical communication devices with exceptional performance. By exploring the unique properties of nonlinear material LiNbO_3_ and photonic crystal multilayer structures, our research has laid the foundation for the development of future devices that exhibit strong coupling, minimal loss, and high efficiency in second-harmonic generation.
